# Adrenal Cushing’s syndrome in children

**DOI:** 10.3389/fendo.2023.1329082

**Published:** 2023-12-12

**Authors:** Valentina Guarnotta, Fabrizio Emanuele, Riccardo Salzillo, Carla Giordano

**Affiliations:** Department of Health Promotion, Mother and Child Care, Internal Medicine and Medical Specialties, Section of Endocrinology, University of Palermo, Palermo, Italy

**Keywords:** hypercortisolism, pediatric, childhood, adrenal tumors, adrenal hyperplasia

## Abstract

Adrenal Cushing’s syndrome is a rare cause of endogenous hypercortisolism in neonatal and early childhood stages. The most common causes of adrenal CS are hyperfunctioning adrenal tumours, adenoma or carcinoma. Rarer causes are primary bilateral macronodular adrenal hyperplasia (PBAMH), primary pigmented adrenocortical disease (PPNAD) and McCune Albright syndrome. The diagnosis represents a challenge for clinicians. In cases of clinical suspicion, confirmatory tests of hypercortisolism should be performed, similarly to those performed in adults. Radiological imaging should be always combined with biochemical confirmatory tests, for the differential diagnosis of adrenal CS causes. Treatment strategies for adrenal CS include surgery and in specific cases medical drugs. An adequate treatment is associated to an improvement of growth, bone health, reproduction and body composition from childhood into and during adult life. After cure, lifelong glucocorticoid replacement therapy and endocrine follow-up are required, notably in patients with Carney’s complex disease.

## Introduction

Cushing’s syndrome (CS) is characterized by an excess of glucocorticoids and represents a diagnostic and therapeutic challenge for clinicians. The total incidence of CS is approximately 0.7-2.4 new cases per million people per year. Of these, only about 10% occur in paediatric age (1). Paediatric and adolescent CS is quite rare, especially when compared to other disorders of this age including growth, puberty, thyroid and metabolic diseases.

The aim of this review is to discuss the causes of adrenal CS and their pathogenesis, focusing on the clinical presentation, diagnostic approach and therapeutic management, showing advantages and disadvantages in a critical way.

## Epidemiology and classification

As in adult, paediatric and adolescent CS can result from an exogenous or endogenous cause. Exogenous CS, probably underdiagnosed, generally occurs in children who need to take steroid therapy. Although the majority of cases result from administration of oral or parenteral glucocorticoids, in childhood the topical and inhaled administration of supraphysiological doses requires particular attention. Since they have a thinner dermis layer of epidermis than adults, children are more vulnerable to systemic effects of topically glucocorticoids. In iatrogenic CS, it is essential for clinicians to be aware of sudden withdrawal of steroids and to inform patients and/or caregivers to prevent adrenal crisis.

Endogenous CS can be divided into ACTH dependent forms such as ectopic CS and pituitary ACTH-secreting tumours or ACTH independent forms including adrenocortical tumours (adenoma or carcinoma) and primary adrenocortical hyperplasia such as primary pigmented nodular adrenocortical disease (PNAD), primary bilateral adrenal macronodular hyperplasia (PBAMH) and McCune-Albright syndrome.

A bimodal age distribution has been reported, with higher incidence of adrenal forms before 7 years and ACTH-dependent forms over 7 years (2–4). Ectopic CS is very rare in children and adolescents with a prevalence <1% although a recent systematic review reported a median prevalence of 7% (X). Cushing’s disease (CD) accounts for 75%, while adrenal causes of CS account for 15% of all cases of hypercortisolism in children.

## Pathogenesis of adrenal causes of CS

The most common causes of adrenal CS are hyperfunctioning adrenal adenoma or carcinoma. Rarer causes are BAMH, PPNAD and McCune Albright syndrome.

## Adrenocortical tumours

Adrenocortical tumours (ACTs) are very rare in childhood although they are an important cause of CS in this age. The reported incidence is about 0.2-0.3% of all paediatric tumours and a very low percentage of them are adrenocortical carcinomas. The incidence is different depending on the geographic area, with a higher incidence in Brazil compared to the United States (5, 6).

Paediatric adrenocortical tumours notably affect children aged from 0 to 4 years old and adolescents. In addition, a gender difference has been reported, with a female predominance before 3 years and after 13 years (5, 6). Two large studies have collected paediatric adrenocortical cancer cases, the International Pediatric Adrenocortical Tumor Registry (IPACTR) (7), which includes 254 patients, and a Brazilian monocentric study including 73 children (8).

The higher incidence in Brazil is explained by a high prevalence of p53 tumour suppression (TP53) mutation, which is involved in ACT pathogenesis. In patients without TP53 mutation, 11p15 chromosome defects have been reported (Beckwith-Wideman syndrome) (6). ACTs are generally functional and are only associated with virilizing signs and symptoms including pubic hair, faster growth and skeletal maturation and external genitalia enlargement in about 50-60% of children (9–12). In some cases virilization is combined with hypercortisolism (about 30% of cases) (13). Less frequently (10-15% of all cases) ACTs tend to present with CS, even though CS secondary to adrenocortical cancer is more frequent in adolescents and is associated with a poor survival rate (3, 14). Less than 5% of patients show feminization or hyperaldosteronism (13).

## Primary adrenocortical hyperplasia

### Primary pigmented adrenocortical disease

PPNAD can be isolated or associated with Carney’s complex. It frequently occurs in adolescents, even though several cases have been described in childhood (15). PPNAD is characterized by black adrenocortical micronodules located in the adrenal gland that appears atrophic in the areas not involved by nodules. Further, non-pigmented adrenocortical nodular disease has also been described, characterized by a unilateral adrenal tumour and absence of pigmentation, caused by a myosin heavy chain 8 mutation (16, 17).

PPNAD is generally caused by a mutation of the protein kinase 1 regulatory subunit 1A (PRKAR1A) gene, leading to activation of the cAMP/PKA pathway associated with a glucocorticoid hypersecretion (18). However, second-line molecular events are involved in cortisol hypersecretion as shown in patients with armadillo-repeat containing 5 (ARMC5) gene mutations (19).

PPNAD is generally characterized by overt CS; it is rarely associated with a subclinical or cyclical CS (20–22). The clinical presentation can rarely be classical (23) and is more frequently characterized by hyperandrogenism and osteoporosis beyond typical symptoms of Carney complex, which can be combined, such as skin pigmentation, cardiac myxomas and endocrine and non-endocrine tumours (15).

### Primary bilateral macronodular adrenal hyperplasia (former name ACTH-independent macronodular adrenal hyperplasia AIMAH)

Primary bilateral macronodular adrenal hyperplasia (PBMAH), is a bilateral adrenal hyperplasia characterized by large yellow-to brown cortisol secreting nodules, not associated with other disorders. It is very rare in childhood and adolescence, while it is more frequent in older patients. In pediatric/adolescent cases, PBMAH can be associated with dominantly inherited genetic condition. It can be sporadic or associated with genetic mutations, such as hyperexpression of the G-protein aberrant receptors and pathogenic variants of MC2R, GNAS, PRKAR1A, and PDE11A. However, the ARMC5 gene is believed to be a major genetic cause of PBMAH, accounting for more than 80% of the familial forms (24). In patients with an aberrant expression of the gastric inhibitory polypeptide receptor (GIPR) in the adrenal glands, cortisol hypersecretion can be food-dependent (25). Clinical presentation can be overt or subclinical CS (26).

### McCune-Albright syndrome

McCune-Albright syndrome, a sporadic heterogeneous disorder caused by somatic or post-zygotic activating mutations in the α subunit of G protein (Gsα gene) is the main cause of CS in the neonatal period. McCune-Albright syndrome is generally characterized by peripheral precocious puberty, *café-au-lait* skin pigmentation and polyostotic fibrous dysplasia, which can be combined with other endocrine disorders including CS, hyperthyroidism and growth hormone excess (27). In addition, it generally presents with severe CS (28–30), typically in the neonatal period, and though severe, it could be also transient. However, milder cases have also been reported (31).

## Clinical presentation

The clinical presentation of paediatric CS is characterized by many different symptoms. Contrary to what is observed in pediatric CD where there is a male prevalence, non-gender differences are observed in pediatric adrenal CS.

**Figure 1:**
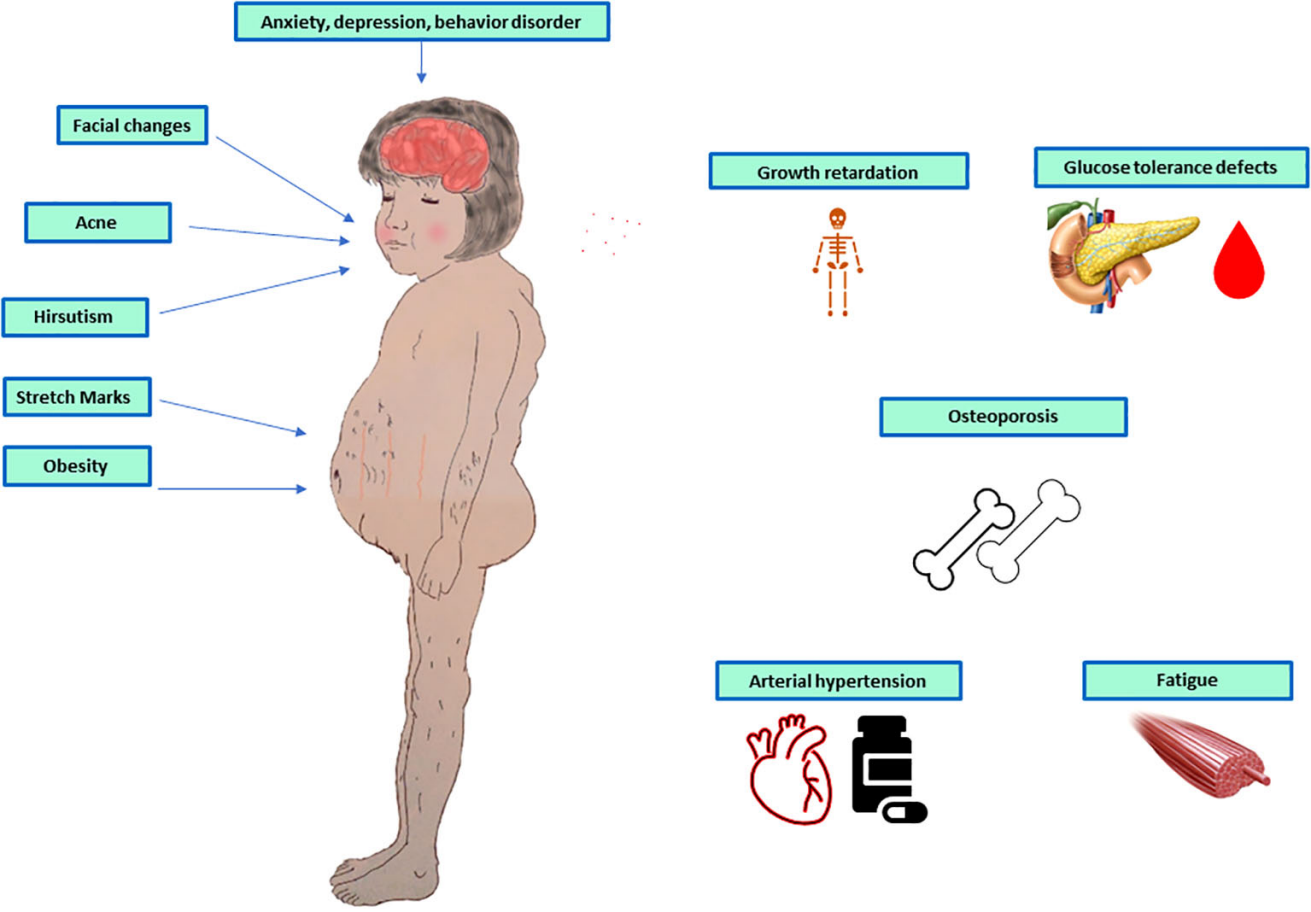
Symptoms and signs of paediatric adrenal Cushing’s syndrome.

The two most common are weight gain and growth retardation. Other signs include faciotruncular fat distribution, skin fragility, arterial hypertension, violaceus skin striae, acanthosis nigricans, hypertensive encephalopathy (32), skin infection, recurrent infection (33), nephrocalcinosis and kidney stone (34), delayed bone mineralization and puberty (35), depression, fatigability, behaviour disorders (36), ion disorders, such as hypokalaemia, and hypercalcemia, glucose intolerance or diabetes, as shown in **Figure 1**.

In a recent retrospective study on a Chinese paediatric population with Cushing’s disease, growth retardation, weight gain, hirsutism and acne were the main clinical signs (37).

Chronic exposure to glucocorticoids results in an overall inhibition of the somatotropic axis with reduced production of GH and IGF-1. This factor, in association with some direct effects of glucocorticoids such as inhibition of collagen synthesis, cartilage sulphation and chondrocyte mitosis, contributes to growth arrest (38). Moreover, prolonged exposure to excess glucocorticoids can lead to pathological reduction of bone mass and in some cases osteoporosis, even in childhood. Another very significant aspect is pubertal development: a pseudo-precocious puberty and virilization were observed with gonadotrophin levels subnormal by a suppressive effect of chronic hypercortisolism.

In a large Brazilian study in 254 patients younger than 20 years of age with newly diagnosed or previously treated ACTs, Michalkiewicz et al. (7) reported a significant frequency of virilization (around 84% in the examined population) as a clinical onset sign in paediatric adrenal CS, either isolated or combined with other symptoms. Isolated Cushing’s syndrome was rare.

The impact of CS on the psychological sphere of children can be much more devastating. Indeed, in adults an improvement in quality of life and cognitive functions are generally described, while in children a worsening of cognitive functions and the appearance of psychopathological manifestations are observed even after the remission of hypercortisolism (39, 40).

Anyway, the clinical presentation of CS is different according to the age onset. In neonatal age it is generally associated with the classical skin marks; bone dysplasia and early puberty can also occur (41). In childhood the common causes of hypercortisolism are ACTs and the most frequent symptoms are hyperandrogenism, hirsutism and acne (early puberty). Another cause is PPNAD, which is not systematically associated with delayed growth (42, 43). In the peri-puberty period the typical symptoms are slow growth and weight gain, combined with delayed puberty, and behaviour disorder (aggressiveness, fatigability, poorer tolerance of physical exercises) (36).

## Diagnosis

### Biochemical exams

After exogenous causes are excluded, a suspect pediatric patient should be referred to a third-level centre. Since there are no pediatric guidelines, the diagnosis and confirmation of hypercortisolism should be performed according to current guidelines for adult CS although there may be some difference (44, 45). It is important to note that none of these tests have not been used extensively in children population.

Three consecutive 24-hour urine collection, to evaluate urinary free cortisol (UFC), corrected for body surface area, are the first-level exams in older children (44). In younger children an adequate 24h urine collection could be difficult, leading to false negative results. False positive results could be obtained in cases of excessive water intake (more than 5L/day), severe obesity, anorexia, malnutrition, and physical and emotional stress, which could be indicators of pseudo-Cushing states (46).

The loss of circadian rhythm represents an hallmark of CS. Late-night salivary cortisol can be easily performed in children, notably in younger children and babies, even though it is characterized by greater inter-laboratory variability and normal levels in children are uncertain (47–49). Recently, in 320 healthy children (174 girls) between 4 and 16 years of age, bedtime and morning salivary cortisol and cortisone were measured by LC-MS/MS (49). The cutoff level for bedtime salivary cortisone was 12.0 nmol/L and salivary cortisol was 2.4 nmol/L, slightly lower than that of adults using the same method (2.8 nmol/L) supporting that age and gender-specific cutoff levels are not required for bedtime salivary cortisol or cortisone. Salivary cortisone and cortisol can be used interchangeably. In addition, exogenous hydrocortisone-contaminated samples can easily be identified by LC-MS/MS because cortisone/cortisol ration is very low.

An overnight suppression test can be performed by administering 25 μg/kg of dexamethasone at 11 p.m./midnight (maximum dose 1 mg) and serum cortisol measurement on the following day at 8 AM (50). Another biochemical test is the 2mg-2days dexamethasone suppression test (DST), which consists in the administration of 20-30 μg/kg/day of dexamethasone (maximum dose 2 mg/day) divided into 0.5 mg doses every 6 h, given at 09.00, 15.00, 21.00 and 03.00 h for 48 h (51, 52). The cortisol cut-off level should be <1.8 ug/dl (50 nmol/L).

**Figure 2:**
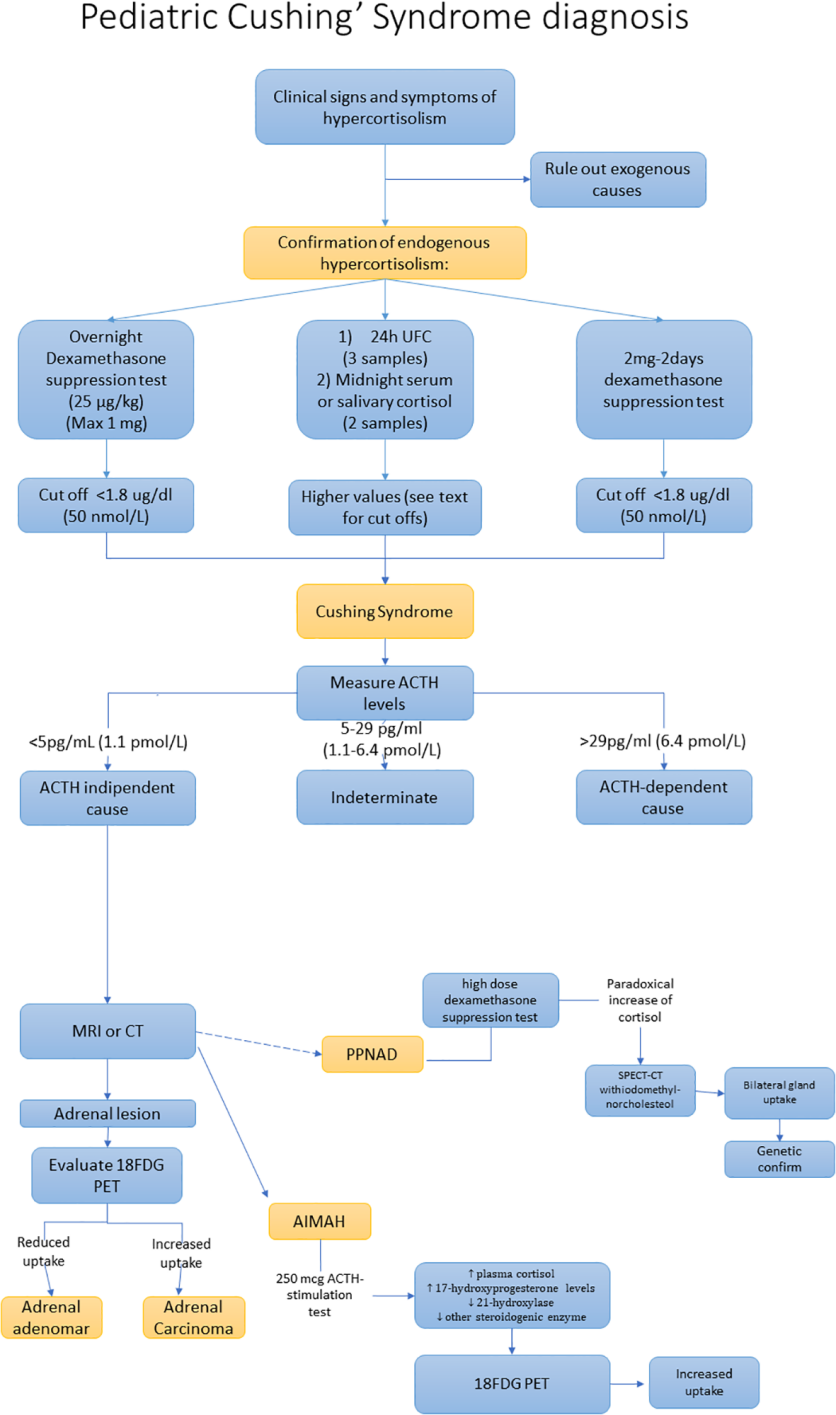
Diagnostic work-up of adrenal Cushing’s syndrome.

Once the diagnosis is confirmed we should distinguish between ACTH dependent and ACTH independent forms, by assaying ACTH levels. If ACTH value is less than 5 pg/mL (1.1 pmol/L), it is strongly indicative of ACTH-independent CS. ACTH levels between 5 and 29 pg/mL (1.1-6.4 pmol/L) can be considered a grey zone, while ACTH values greater than 29 pg/mL (6.4 pmol/L) are strongly suggestive for ACTH-dependent conditions. See diagnostic work-up in **Figure 2** (53).

Additional exams are the high dose dexamethasone suppression test (HDDST) which can be performed for the PPNAD diagnosis. Indeed, in patients with PPNAD, HDDST can provide a paradoxical answer, with an increase of UFC/cortisol instead of a reduction. However, the sensitivity of the HDDST is quite low (39%) (12, 54, 55). Recently some studies identified a cut-off of UFC post HDDST/UFC pre HDDST of 1.08 reporting a sensitivity of 84% and a specificity of 75.6% (56). In patients with a high suspicion for PPNAD, the genetic analysis to identify mutations of the PKAR1A could confirm the diagnosis of PPNAD, notably in patients with combined Carney’s complex.

Modest elevations in plasma 17-hydroxyprogesterone or urinary 17-OH corticosteroids levels are frequently found in PBAMH (57, 58). In these patients, the 250 mcg ACTH-stimulation test leads to increased plasma cortisol and 17-hydroxyprogesterone levels reflecting the large hyperplastic adrenal glands with relative 21-hydoxylase and other steroidogenic enzyme deficiencies (57, 59). However, the suppressed ACTH levels allow one to distinguish PBAMH from congenital adrenal hyperplasia.

### Radiological imaging

Adrenal imaging is essential for the definition of the adrenal CS cause. For the differential diagnosis, an upper abdomen ultrasound, which can be considered the first-line radiological exam, can be performed, followed by adrenal CT or MRI, which can show more clearly a single adrenal tumour or bilateral hyperplasia.

Adrenal CT scan and MRI show higher sensitivity and accuracy than ultrasound and ensure a better definition of fat richness, providing information on size, calcifications and heterogeneity of the lesion, and the anatomical relationship with adjacent organs. A CT scan using 3 mm or less in thickness can reveal micronodules inside the adrenal gland (60). MRI shows several advantages compared to CT scan, including absence of ionizing radiation, capability of imaging multiple planes, and improved tissue contrast differentiation. For this reason, an MR scan should be preferred in patients who need long-term follow-up.

High-resolution 18FDG PET could be useful to distinguish benign from malignant tumours (61, 62). However, an increased uptake of radiotracer has been reported in PBAMH, with an uptake similar to that of a malignant lesion (maximum standardized uptake value > 3.1), reflecting the high glycolytic activity of nodular lesions (63). Single-photon emission computed tomography (SPECT-CT) with the iodomethyl-norcholesterol (I-131) radiotracer can be useful in patients with PPNAD, showing bilateral gland uptake (64). However, considering the high radioactive exposure, the use of this method should be reserved for exceptional cases.

### Treatment

The therapeutic management of adrenal causes of CS depends on the primary cause. Adrenalectomy is the first-choice treatment. However, when surgery is contraindicated or refused, medical therapy should be recommended.

Unilateral adrenalectomy is indicated for unilateral benign cortisol-secreting adenomas.

Surgery is also the first-line therapeutic approach for adrenocortical cortisol-secreting cancers (10, 65). Contrasting findings are currently available on the usefulness of lymph node dissection (66). Mitotane is often used in adults as adjuvant therapy, but due to its neurotoxicity, in children it should be used with caution (67, 68). A nonrandomized single-arm study showed better survival in those children who achieved a mitotane level greater than 14 mg/L (69). In patients with unresectable adrenocortical cancers adjuvant chemotherapy could be used. Chemotherapy in children generally includes cisplatin and etoposide with or without doxorubicin combined with mitotane. Despite this multimodality approach, prognosis of paediatric adrenocortical cancer with metastatic disease remains poor, with an estimated 5-year survival below 20% (70, 71).

In cases of adrenocortical cancers with distant metastases, checkpoint inhibitors could be recommended. Notably, some encouraging findings have been reported with pembrolizumab (10, 72).

Bilateral adrenalectomy is strongly recommended in patients with PPNAD (73). Surgery is associated with biochemical remission, growth catch-up, weight loss and improvement of CS phenotype (74). Unilateral adrenalectomy can be associated with remission of symptoms without risk of adrenal insufficiency. However, in some patients recurrence of CS could occur (75, 76).

In patients with PBAMH the suggested treatment consists in surgical removal of one or both adrenal glands, and rarely in specific drugs inhibiting excessive cortisol secretion (77, 78). The best surgical approach is still on debate. Indeed, unilateral adrenalectomy has a remission rate of hypercortisolism reaching 96%, but with a recurrence rate of 23% (75, 79, 80). However, a post operative follow-up is strongly required in order to prevent adrenal insufficiency and CS recurrence (81, 82).

In patients with contraindications or refuse of surgery, drugs inhibiting adrenal cortisol synthesis (ketoconazole, metyrapone and recently osilodrostat) have been used in rare cases (83). At the moment, there are no medications approved by EMA or FDA for use in pediatric CS.

Recently, a child with mild, cyclical CS due to PBAMH, who carries a novel germline pathogenic variant in KCNJ5, was treated at 4 years 9 months with very low doses of ketoconazole (300 mg/day), increased to 400 mg/day at 8 years. She showed improved linear growth and normalization of BMI, along with resolution of behavior changes and normalization of blood pressure. However, the discontinuation of the drug for 6 weeks led to recurrence of CS (84).

Osilodrostat has not yet been approved for pediatric patients, but is currently being evaluated in a small Phase II trial (NCT03708900) in the pediatric population (<18 years) and the results are expected at the end of 2023. Recently, in a 14-year-old male with ectopic CS due to metastatic pancreatic tumor, osilodrostat (18 mg twice daily) was well tolerated and obtained a rapid improvement and normalization of UFC (85).

Glucocorticoid replacement therapy has a significant role in the management of patients with adrenal CS who have undergone adrenalectomy. Generally glucocorticoid replacement therapy should be recommended in the pre- and perioperative periods and after surgery for at least 6 months from the adrenalectomy (1). In patients who have undergone bilateral adrenalectomy, a chronic steroid replacement therapy is required, except for patients who have undergone adrenal sparing surgery with preservation of healthy cortical tissue.

## Conclusions

Adrenal CS is a quite rare condition in childhood that occurs more frequently in children aged less than 7 years. Adrenal CS can result from adrenocortical tumours including adenoma or carcinoma or from primary adrenal hyperplasia including PPNAD, PBAMH or McCune Albright syndrome. The clinical presentation can vary according to the age of onset. However, weight gain, clinical hyperandrogenism and growth disorders are the most frequent signs and symptoms. Early diagnosis is essential to improve clinical symptoms and to better manage the primary cause. Indeed, in cases of adrenocortical cancer an early diagnosis is associated with a better survival. Unilateral or bilateral adrenalectomy is the first-line therapeutic approach. In patients who have undergone bilateral adrenalectomy lifelong glucocorticoid replacement therapy is required. The prognosis is generally good, except for patients with adrenocortical cancer, for which it depends greatly on the initial stage.

## Author contributions

VG: Conceptualization, Writing – original draft, Writing – review & editing. FE: Writing – original draft. RS: Writing – original draft. CG: Conceptualization, Supervision, Writing – review & editing.
